# *Cunninghamella echinulata* DSM1905 biofilm-based L-asparaginase production in pneumatically-driven bioreactors

**DOI:** 10.1371/journal.pone.0308847

**Published:** 2024-09-20

**Authors:** Romeu Cassiano Pucci da Silva Ramos, Nicoly Subtil de Oliveira, Luiz Fernando Bianchini, Luciana Reis Azevedo-Alanis, Ida Chapaval Pimentel, Ana Maria Trindade Gregio Hardy, Ramiro Mendonça Murata, Jarka Glassey, Edvaldo Antonio Ribeiro Rosa

**Affiliations:** 1 Graduate Program in Dentistry, Pontifícia Universidade Católica do Paraná, Curitiba, Brazil; 2 Xenobiotics Research Unit, Pontifícia Universidade Católica do Paraná, Curitiba, Brazil; 3 Graduate Program in Animal Sciences, Pontifícia Universidade Católica do Paraná, Curitiba, Brazil; 4 Department of Microbiology, Immunology and Parasitology, Federal University of Paraná, Curitiba, Brazil; 5 College of Pharmacy and Pharmaceutical Sciences, The University of Toledo, Toledo, Ohio, United States of America; 6 The Brody School of Medicine, East Carolina University, Greenville, North Carolina, United States of America; 7 School of Engineering, Newcastle University, Newcastle-upon-Tyne, United Kingdom; Konkuk University, REPUBLIC OF KOREA

## Abstract

We evaluated by comparing the performance of three pneumatically-driven bioreactors in the production of L-asparaginase (L-ASNase), an enzyme used to treat leukaemia and lymphoma. A two-step screening process was conducted to detect *Cunninghamella* spp. strains producing L-ASNase. *Cunninghamella echinulata* DSM1905 produced the highest levels of L-ASNase during screening assays. Subsequently, fermentations were performed in bubble column (BCR), airlift (ALR), and hybrid fixed-bed airlift (FB-ALR) bioreactors to determine the best upstream bioprocess. Mycelial biomass production was higher in BCR than in ALR and FB-ALR (p ≤ 0.0322). The activity of L-ASNase produced in FB-ALR, in which the fungus grew as a consistent biofilm, was significantly higher (p ≤ 0.022) than that from ALR, which was higher than that of BCR (p = 0.036). The specific activity of ALR and FB-ALR presented no differences (p = 0.073), but it was higher than that of BCR (p ≤ 0.032). In conclusion, *C*. *echinulata* DSM1905, grown under the biofilm phenotype, produced the highest levels of L-ASNase, and FB-ALR was the best upstream system for enzyme production.

## Introduction

L-asparaginase (L-ASNase) is a chemotherapeutic drug that treats acute lymphocytic leukaemia (ALL), acute myeloid leukaemia (AML), and some types of non-Hodgkin’s lymphoma [1] by catalysing L-asparagine into L-aspartate and ammonia.

L-asparagine is a non-essential amino acid produced by normal cells by the action of the constitutive enzyme asparaginase synthetase. Lymphatic tumour cells cannot synthesise some amino acids, forcing the capture of free molecules from the surrounding environment, including L-asparagine. Therapeutic L-ASNase hydrolyses free L-asparagine, limiting its availability to the tumour cells and consequently inhibiting their development [2].

Industrially, this enzyme is produced by fermentation using the Gram-negative bacillus *Erwinia chrysanthemi* or other Enterobacteriaceae [3, 4]. However, bacteria-derived enzymes can provoke hypersensitivity reactions [5], and due to their L-glutaminase-like activity, they can cause severe adverse effects [6]. L-ASNase variants with less adverse effects have been discovered in eukaryotic microorganisms, especially filamentous fungi [7–9].

Solid-state fermentation (SSF) and submerged fermentation (SmF) [10, 11] have been proposed as suitable methods for the bulk production of L-ASNase when using fungi. SmF advantages include better process control and more straightforward downstream stages. Bioreactors used for SmF may present many variations, configurations, and architectures. The most popular are the stirred tanks (STR) and the pneumatically impelled.

Pneumatically impelled bioreactors cause lower shear stress, maintaining the fungal mycelial structures. They also present good heat and mass transfer coefficients and lower energy inputs when compared to popular STR. However, to our knowledge, no evaluations amongst different pneumatically impelled bioreactors in the production of fungal L-ASNase have been carried out, which justifies the conduct of this study.

## Material and methods

### Fungal strains

L-ASNase secretory activity was evaluated for *Cunninghamella blakesleeana* DSM1906, *C*. *echinulata* DSM1905, *C*. *elegans* DSM1908, *C*. *elegans* DSM8217, and *C*. *elegans* DSM63299. Such *Cunninghamella* spp. strains were kindly provided by Prof. Cormac Declan Murphy, School of Biomolecular and Biomedical Science, University College (Dublin, Ireland).

### Qualitative L-ASNase production screening

Sabouraud Dextrose Agar (SDA) was inoculated and incubated at 28°C for 120 h. Mycelial disks (5 mm Ø) were individually transferred to the central area of 90 mm Petri dishes containing 10 mL of modified Czapek Dox medium [CDM; 2 g L^-1^ glucose, 10 g L^-1^ L-asparagine, 1.52 g L^-1^ KH_2_PO_4_, 0.52 g L^-1^ MgSO_4_.7H_2_O, 0.52 g L^-1^ KCl, 0.001 g L^-1^ CuNO_3_.3H_2_O, 0.001 g L^-1^ ZnSO_4_.7H_2_O, and 0.001 g L^-1^ FeSO_4_.7H_2_O with 2% agar and 0.009% phenol red (pH 6.2)] [7]. SDA without L-asparagine served as control. The dishes were incubated at 30°C for 120 h. Reddish zones formed around the fungal colonies indicated possible production of L-ASNase. Dishes with modified CDM-agar containing 10 g L^-1^ L-glutamine instead of L-asparagine were used to verify L-glutaminase production (L-GLNase). They were carried out nine repetitions per strain.

Colonies’ and halos’ diameters were measured using a digital calliper in the N-S, E-W, Ne-Sw, and Nw-Se cardinal orientations. Average measurements (four orientations × nine repetitions) were calculated and transformed into area values. Presumptive enzyme activity was calculated for each strain according to Eq 1, where HA is the colony plus halo area (hydrolysis area), and CA is the area diameter (1-Pz value). Higher enzyme activity represents greater hydrolytic activity [12]. Assays were performed in nine replicates, and average values for each strain were obtained and used further.


$$Enzyme\;activity=1-(\frac{HA}{CA})$$ (1)

### Quantitative L-ASNase production screening

After qualitative assays, the strains were cultivated in SDA at 30°C. After 7 d, 2 mL of 0.05% Tween^®^20 were added to the dish and gently rounded for 2 min to collect fungal spores. One millilitre aliquot from each strain was collected and transferred to 250 mL conical flasks containing 50 mL of CDM-broth without a pH indicator. Cultures were maintained at 120 rpm and 30°C for 120 h. Supernatants were collected and immediately evaluated for hydrolytic activity using the Nessler assay.

Reaction mixture I consisting of 50 μL of 50 mM Tris-HCl buffer (pH 7.2), 200 μL of 40 mM L-asparagine, and 50 μL of the sample was incubated at 37°C for 60 min. Blank for substrate controls consisted of 300 μL of 50 mM Tris-HCl buffer. The reaction was stopped by adding 50 μL of 1.5 M TCA.

Reaction mixture II consisted of 187.5 μL of _dd_H_2_O, 25 μL of reaction mixture I, and 37.5 μL of Nessler’s reagent. Reaction mixtures were incubated for 20 min at 25°C. Absorbances were measured at 450 nm using a microplate reader. Ammonium sulphate was used to build a standard curve, and the assays were performed in decuplicate. One unit of L-ASNase was defined as the amount of enzyme in 1 mL that catalyses the formation of 1 μmol of ammonia per minute at 37°C, in pH 7.2.

Fungal biomasses were determined by weighing. The mycelia were dried on pre-weighed #3 Whatman filter paper disks at 80°C for 48 h. The dry biomass of each strain was determined as shown in Eq 2, where PF is the mass of dry filter paper disk without mycelia and PFM is the mass of filter paper plus mycelium

Drybiomass=PFM–PF (2)

The specific enzyme activity was determined by dividing the enzyme activity by the dry fungal biomass.

### Bioreactors

**Fig 1:**
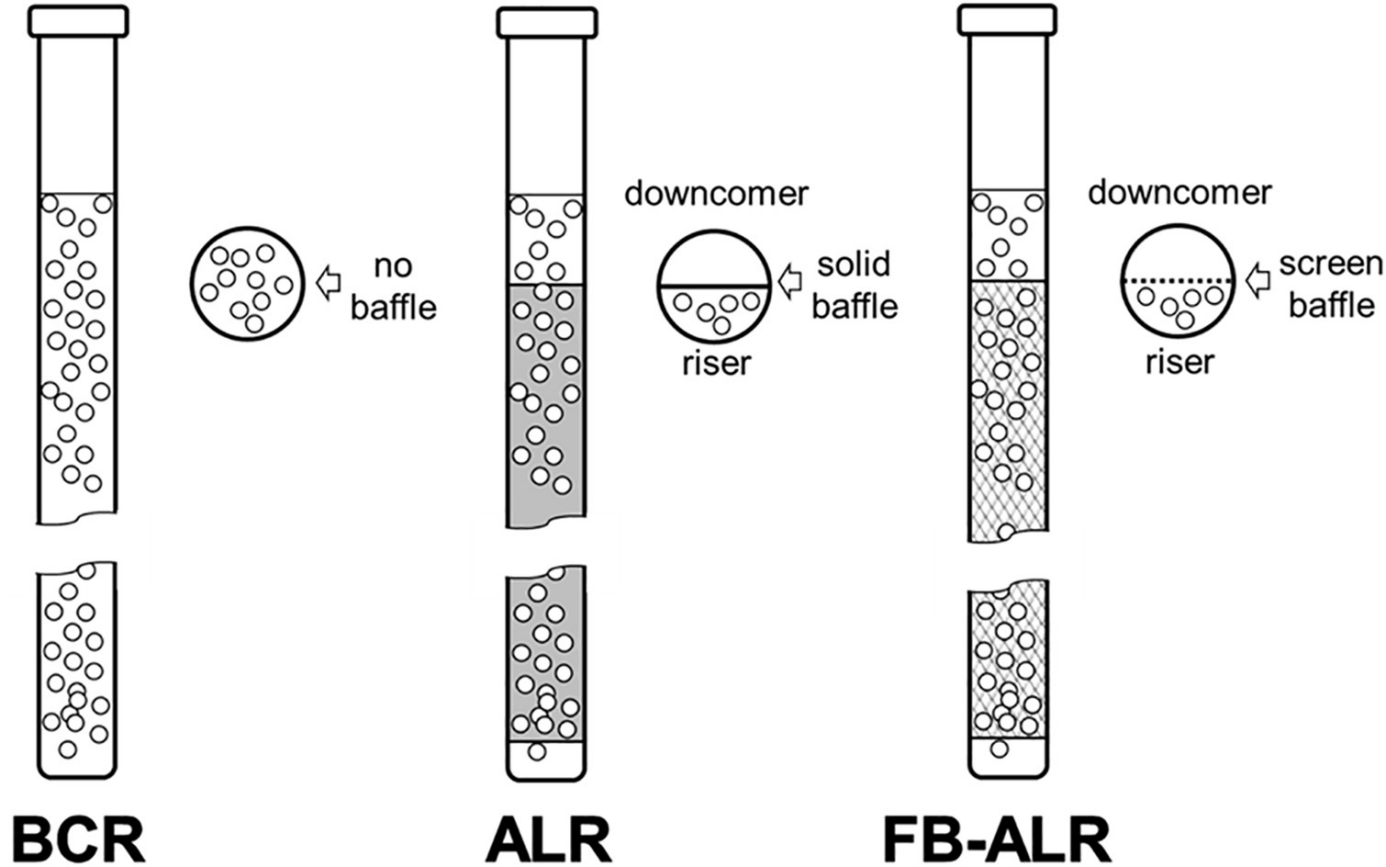
Bioreactor geometries. BCR = Bubble column reactor; ALR = Airlift reactor; FB-ALR = Fixed bed–airlift reactor.

**Fig 2:**
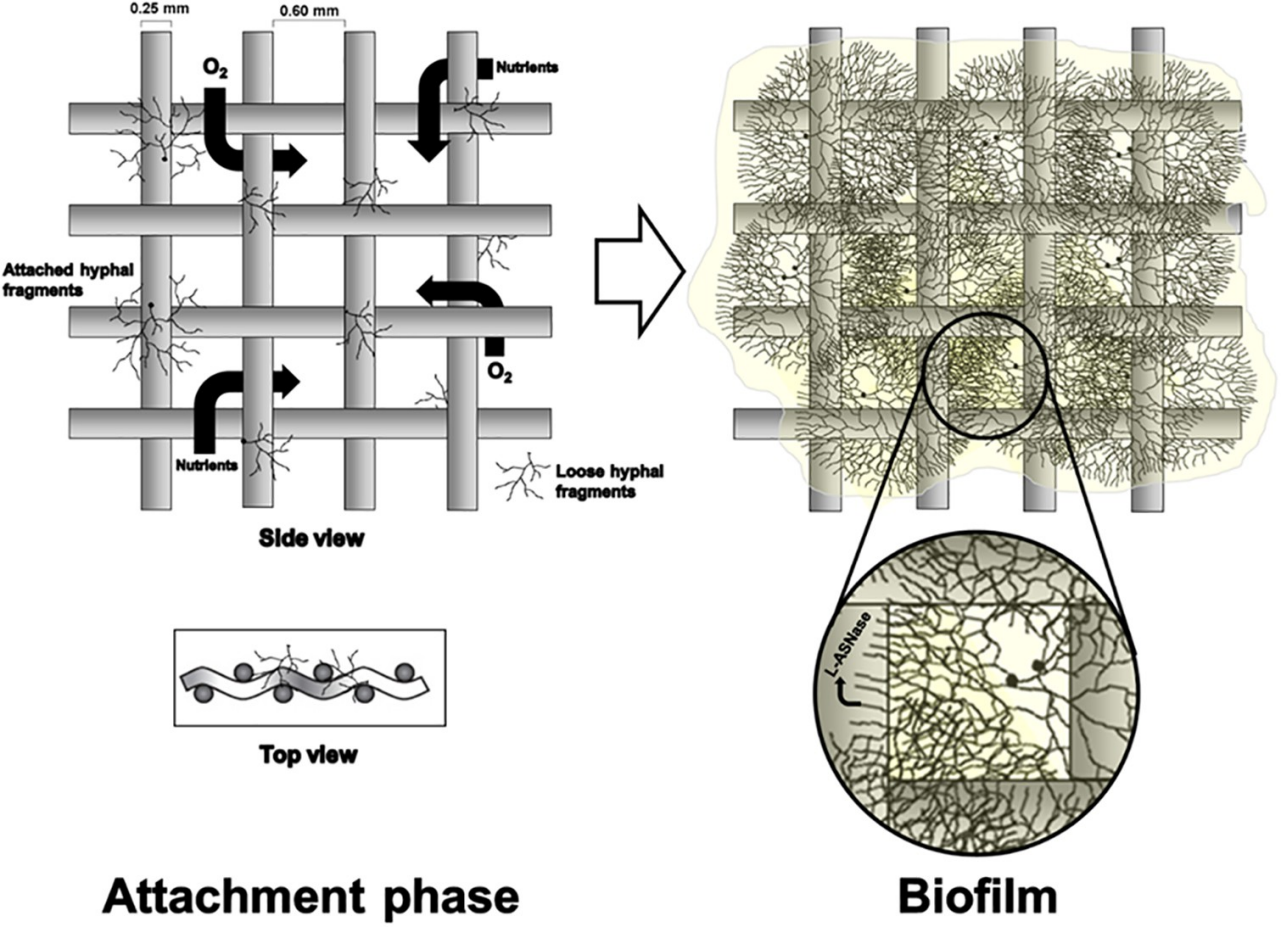
Stainless steel mesh baffle and proposed *C*. *equinulata* biofilm formation mechanism in the FB-ALR.

Cylindrical borosilicate glass vessels [750 mm (h) × 55 mm (Ø)] were operated as bubble column reactors (BCR), airlift reactors (ALR), and hybrid fixed bed-airlift reactors (FB-ALR), as represented in Fig 1. ALRs had a split-internal loop geometry and internal baffles built with 500 (h) × 55 (w) × 2 mm (t) glass sheets and 10 mm annular cutouts at the bases. FB-ALRs had the same geometry as the ALRs, but the baffles [500 (h) × 55 (w) × 0.5 mm (t)] were built using AISI304 stainless steel mesh (0.60 mm) made with 0.25 mm Ø wire. These baffles served as anchor points (scaffolds) for the growth of fungal biofilms. This anchoring prevents biomass accumulation on the liquid surface, obstructing gas exchange and causing gas flooding (Fig 2).

Our group’s previous study presented details of bioreactors’ architecture and hydrodynamic and mass transfer parameters [13].

### Preparation of *C*. *echinulata* DSM1905 starter

Preliminary qualitative/quantitative screening showed that *C*. *echinulata* DSM1905 was the best strain-producing L-ASNase; hence, it was selected for SmF assays.

Five Petri dishes (90 × 15 mm) containing SDA were inoculated and incubated at 28°C for 120 h. All fungal biomass obtained was scrapped and transferred to a 40 mL Tenbroeck homogeniser. The disintegrated biomass was diluted in sterile H_2_O, and the cell concentration was turbidimetrically adjusted to the #5 tube of the McFarland scale.

### Submerged fermentations

Bioreactors containing 800 mL of M3 medium (40 g L^-1^ glucose; 5.55 g L^-1^ K_2_HPO_4_; 1.7 g L^-1^ KH_2_PO_4_; 2.1 g L^-1^ NH_4_Cl; 0.2 g L^-1^ MgSO_4_.7H_2_O; 0.01 g L^-1^ FeSO_4_.7H_2_O; 0.03 g L^-1^ CaCl_2_.2H_2_O) were loaded with 15 mL of *C*. *echinulata* DSM1905 inoculum and 10 μL of antifoam silicone emulsion (Biotec Ltd, Cotia, Brazil).

Biomass production occurred at 28°C and 0.5 vvm of sterile atmospheric air. After 96 h of growth, 2.5 mL of 1.836% L-asparagine were added, and the fermentations continued for 48 h. All assays were performed in decuplicate.

As previously described, biomass and enzyme activities were determined for the three bioreactors. Ten repetitions of tests were carried out in each bioreactor.

### Statistics

The results obtained for the quantitative L-ASNase production screening and the different bioreactors were submitted for verification of normal distribution by the Kolmogorov-Smirnov test. As the dependent variables showed normal distribution, one-way ANOVA was performed. When there was an indication of the difference between at least two bioreactors, a two-to-two comparison was performed using the Tukey HSD test for homogeneous variances. The power of the test was 94.97%, and the significance level adopted in all tests was 5% (p < 0.05).

## Results

### Screening tests

Although numerical results were assessed, qualitative assays were carried out to confirm the production of L-ASNase and to exclude possible L-GLNase strains. All strains were classified as L-ASNase positive and L-GLNase negative, as presented in Table 1.

**Table 1:** Colonies’ areas (CA) and hydrolysis’ halos (HA) for L-asparaginase (L-ASNase) and L-glutaminase (L-GLNase) obtained during screening assays*.

Strain	L-ASNase				L-GLNase			
	CA (mm^2^)	HA (mm^2^)	HA/CA (Pz)	1-Pz**	CA (mm^2^)	HA (mm^2^)	HA/CA (Pz)	1-Pz
*Cunninghamella blakesleeana* DSM1906	125.18 ± 48.24	146.02 ± 56.26	0.85	0.15	223.48 ± 12.31	0.00	nc***	nc
*Cunninghamella echinulata* DSM1905	68.20 ± 12.21	262.12 ± 109.04	0.26	0.74	583.20 ± 20.88	0.00		
*Cunninghamella elegans* DSM1908	390.74 ± 47.13	878.78 ± 119.13	0.44	0.56	360.01 ± 5.69	0.00		
*Cunninghamella elegans* DSM63299	722.38 ± 168.62	1241.28 ± 328.22	0.58	0.42	705.91 ± 32.28	0.00		
*Cunninghamella elegans* DSM8217	528.89 ± 33.37	1325.70 ± 69.09	0.40	0.60	544.49 ± 12.15	0.00		

*Nine replicates were carried out per strain × four cardinal orientations.

**Pz is the value obtained dividing CA by HA; the higher the 1-Pz value higher the enzyme activity.

***nc indicates no enzyme activity.

**Fig 3:**
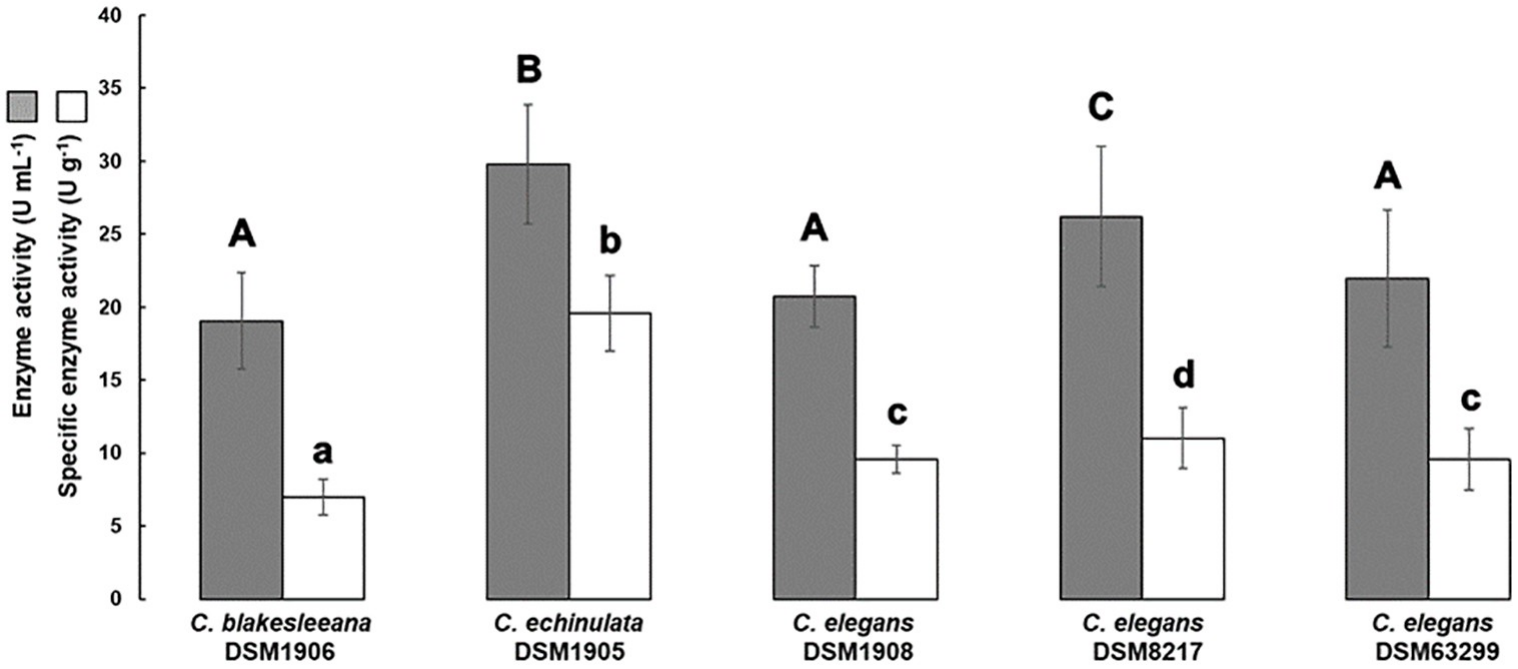
Enzyme activity and specific enzyme activity of L-ASNase for selected fungal strains screened by qualitative tests.

The quantitative screening assay revealed similar L-ASNase enzyme activity for *C*. *blakesleeana* DSM1906, *C*. *elegans* DSM1908, and *C*. *elegans* DSM63299 (19.061 U mL^-1^, 20.749 U mL^-1^, and 21.950 U mL^-1^, respectively; p > 0.500), as presented in Fig 3.

The highest L-ASNase activities were observed in *C*. *echinulata* DSM1905 (29.791 U mL^-1^) and *C*. *elegans* DSM8217 (26.208 U mL^-1^). Regarding specific enzymatic activity, *C*. *echinulata* DSM1905 performed better than the others with 19.581 U g^-1^ (p < 0.01). Therefore, this strain was further chosen for fermentation tests.

### Fermentations

The growth of fungal biofilms was observed in all three bioreactor systems. As expected, dense and well-structured biofilms along the entire length of the scaffolds were observed in the FB-ALR. In the ALR, biofilms were observed in retention areas at the edges of the baffles in addition to non-circulating planktonic mycelial growth that accumulated in the upper portions of the liquid phase. Growth in the form of circulating flocs was verified in the BCRs; however, after the 72^nd^ hour, floating biomasses became denser and accumulated at the bottom of the vessels.

Table 2 shows the results of the fermentations carried out in different bioreactors. No significant differences in biomass production were observed between ALR and FB-ALR (p = 0.068), and both were lighter than those generated in BCR (p ≤ 0.0322).

**Table 2:** Fungal biomass and L-ASNase activity for *C*. *equinulata* DSM1905 after 96-h fermentations.

Bioreactor	Biomass (g)	Enzyme activity (U mL^-1^)	Specific enzyme activity (U g^-1^)
**BCR**	4.93 ± 0.56ª	728.35 ± 80.29ª	149.73 ± 25.10ª
**ALR**	4.23 ± 1.03^b^	855.21 ± 35.08^b^	218.15 ± 76.40^b^
**FB-ALR**	4.21 ± 0.31^b^	1173.59 ± 96.82^c^	279.22 ± 22.61^b^

Similar letters indicate no difference among fermentations (p > 0.05). Different letters indicate differences amongst fermentations (p < 0.05)

The enzyme activity of L-ASNase produced in FB-ALR was significantly higher (p ≤ 0.022) than that from ALR, which was higher than that of BCR (p = 0.036). The specific activity of ALR and FB-ALR presented no differences (p = 0.073), but it was higher than that of BCR (p ≤ 0.032).

## Discussion

Our first goal was identifying *Cunninghamella* strains capable of producing L-ASNase through primary screening in a semi-solid medium. More than one strain capable of producing this enzyme was detected, which was promising.

In functional (quantitative) screening carried out in a liquid culture medium, the greater enzyme production observed here might be due to the greater biomass production, thus making the scale projection difficult. Normalisation with an assured variable, such as dry biomass, wet biomass, metabolic activity, total protein, or total nitrogen, is recommended to avoid this. This study used dry biomass as the normalisation factor for convenience as it is a very reliable variable [14]. Following normalisation, *C*. *echinulata* DSM1905 showed the highest values of specific enzymatic activity, indicating that the zygomycete strain produces more enzyme per unit of dry biomass than the others. It is interesting because, with less biomass, a considerable amount of enzyme can be obtained.

To our knowledge, this is the first study that considered L-ASNase production using *Cunninghamella* spp. Besides being a good L-ASNase producer, *C*. *echinulata* becomes interesting once there is no evidence that it produces known mycotoxins [15–17]. Consequently, no supplementary treatments are required to remove unwanted substances during the downstream processing. Indeed, it has been reported that *Cunninghamella* spp. can biotransform zearalenone, β-zearalenol, and other mycotoxins, causing their inactivation [18].

Therefore, L-ASNase produced by *C*. *echinulata* DSM1905 is preferable for medicinal and veterinary purposes since there is no co-production of L-GLNase, as observed in our screening assays in a semi-solid medium. L-glutamate generated by the L-GLNase action presents many harmful mechanisms that affect neuronal function and viability [19].

The second objective of this study was to determine which pneumatically-driven bioreactor is most suitable for fungal L-ASNase production. Although BCRs present excellent mixing capacities and heat/mass transfer rates [20], they generate a phenomenon known as “liquid phase back mixing,” which incurs low substrate conversion [21]. Backmixing likely promotes the formation of flocs in suspension, which coalesce and form dense aggregates that float and impair gas exchange at the liquid-gas interface, ending with the mycelial mass being deposited at the bottom. We hypothesise that these deposits prevented the access of the inducer (L-asparagine) to the interior of the biomass, with a consequent reduction in enzyme biosynthesis and secretion.

ALRs possess a geometry that drives the cyclic movement of the three-phase solid–liquid–gas system, promoting satisfactory mixing and dissolved oxygen transfer rate (OTR) [22]. However, after 48 h, the massive tangle of hyphae settled at the gas-liquid interface above the baffles, impairing the gas exchange in the headspace and making the regular return of the liquid through the downcomer difficult. This may have somehow limited the L-ASNase production, not allowing the maximum enzyme to be obtained.

FB-ALR allowed the formation of a biofilm tightly adhered to the stainless-steel mesh without accumulation of mycelial biomass at the bottom or the top in the liquid-gas interface. The baffle remained entirely permeable during the initial hours, favouring bubbles’ fragmentation and increasing oxygen dissolution [13]. After completely covering its surface with fungal biofilm (*ca*. 48 h), the bioreactor functioned as a conventional ALR, with the biofilm-trapped cells having homogeneously access to nutrients and oxygen. This effect might have positively influenced the production of L-ASNase as more cells were exposed to the enzyme inducer.

The L-ASNase production in bioreactors (≥ 728.35 U mL^-1^) was robustly higher (p < 0.001) than in conical flasks (6.94 U mL^-1^ to 19.58 U mL^-1^), a common resource employed in screening trials. This disparity is due to the optimised growth and secretory conditions favoured within bioreactors’ environments. Higher oxygen transfer, better heat dissipation, and mixing properties of pneumatically-driven bioreactors enable better cell growth and improved enzyme secretion [23, 24]. On the other hand, in conical flasks, cells grow under a phenotype of planktonic cells that take oxygen from a poor gas-containing headspace [25]. Furthermore, fungal biofilms are more efficient than planktonic cells in secreting enzymes [26–28].

Another approach to enhance L-ASNase secretion involved adding a small amount of inducer L-asparagine after 96 h of growth, followed by more 48 h of fermentation. This strategy has been advocated by other groups that have used inducers with concentrations varying from 0.1% to 2% [29–33], higher than the 0.046% used here. When culture media are formulated with L-asparagine as an original ingredient, it serves as a nutrient consumed during biofilm formation. In our study, the amino acid was added 96 h after inoculation, when mature biofilms were established [34–36], acting as a true inducer. We propose that small amounts of inducer added after biofilms are formed can improve the bioprocess.

Regarding raw L-ASNase production, the results obtained here were higher than those compiled for submerged fermentations [37, 38], which widely varied between 16 U mL^-1^ for the basidiomycete *Flammulina velutipes* [39] until 160 U mL^-1^, for the ascomycete *Penicillium cyclopium* [40]. High values for enzyme activity have been previously reported for the ascomycete *Trichoderma viride* with 650 U mL^-1^ [41], for the zygomycete *Mucor hiemalis* with 1203.55 U mL^-1^ [42], and for *P*. *cyclopium* with 1992 U mL^-1^ [43]. However, in these last studies, the L-ASNase activity was measured after concentration with cold acetone and purification by ion exchange chromatography. In contrast, our enzyme has not received any of such treatments. Therefore, *C*. *echinulata* DSM1905 seems to be a promising strain for L-ASNase production.

Upscaling experiments must be done in a higher volume pilot bioreactor to conclude the upstream step. Downstream procedures shall be carried out to concentrate and purify the L-ASNase produced. Afterwards, further tests for molecule characterisation involving its molar mass determination, isoelectric point (pI), amino acid composition, and activity in human and animal physiological pHs and temperatures must be conducted. To become a candidate for preclinical tests, *C*. *echinulata* L-ASNase must prove its efficacy against leukaemia and lymphoma in cell cultures and safety, neither inducing hypersensibility nor toxicity.

In this study, we rationally selected L-ASNase-producing fungal strains using a two-step screening protocol and determined that *C*. *echinulata* DSM1905 is a good producer. Additionally, hybrid FB-ALR favours the formation of fungal biofilms that can produce L-ASNase.

## Supporting information

S1 Data (XLSX)
